# Effect of Casein Hydrolysates on Intestinal Cell Migration and Their Peptide Profiles by LC-ESI/MS/MS

**DOI:** 10.3390/foods8030091

**Published:** 2019-03-06

**Authors:** Søren D. Nielsen, Stig Purup, Lotte B. Larsen

**Affiliations:** 1 Department of Food Science, Aarhus University, Blichers Allé 20, P.O. Box 50, DK-8830 Tjele, Denmark; lbl@food.au.dk; 2 Department of Animal Science, Aarhus University, Blichers Allé 20, P.O. Box 50, DK-8830 Tjele, Denmark; stig.purup@anis.au.dk

**Keywords:** dairy, wound healing, IEC-6 cells, scratch assay, functional foods, bioactive, peptide

## Abstract

Potential beneficial effects of bioactive peptides derived from casein on epithelial cellular wound healing in the gastrointestinal tract were studied. Bovine casein was digested by a combination of pepsin and pancreatic proteases at different time intervals to represent ranges of duration of gastrointestinal digestion. Intestinal epithelial cells were used as an in vitro model of the small intestine. The effect of casein hydrolysates on cell migration was studied by scratch assay as a model of wound healing. Casein digested by pepsin and pancreatin for 10 to 30 min were found to have a significant stimulatory effect of >40% on cell migration relative to the control. A potential effect of casein gastrointestinal digests on gastro-intestinal wound healing has not previously been reported. The peptide profiles of active as well as inactive casein hydrolysates were characterised by liquid chromatography coupled to ion trap tandem mass spectrometry. By comparison of identified peptides in active and inactive casein hydrolysates, a pool of 11 peptides derived from casein were identified as potential candidates for effects on cell migration. Searching the milk bioactive peptide database (MBPDB) showed that 15 of the identified peptides had known biological functions such as antimicrobial, antioxidant, and immunomodulatory activity.

## 1. Introduction

Apart from its supply of amino acids, nutrients, vitamins, and minerals, milk also contains a range of peptides encrypted in proteins with biological functions, e.g., protection against infection [1] and promoting development of the gut [2]. Bovine colostrum, containing a variety of growth factors and cytokines, has also been found to have a positive effect on maintaining gastro-intestinal health and epithelial integrity, as well as on several gastro-intestinal disorders like inflammatory bowel diseases (IBD) [2]. Studies have shown that bioactive peptides are encrypted within the sequences of both casein and whey proteins [3], and can be released by hydrolysis during gastro-intestinal digestion [4,5]. The bioactivity of a range of casein-derived peptides have been investigated, and specific peptides have been identified with i.e., opioid activities [6], antihypertensive properties [7] or immune modulating effects [8]; the latter covering both immune stimulating and immune suppressive activities [9]. Interestingly, we recently reported that different commercial casein-based milk protein preparations induced intestinal cell migration and proliferation [10]. A recent study further found that a peptide from camel milk could be beneficial towards diabetic wound healing [11]. Taken together, this suggests a possibility for a positive effect of milk protein hydrolysates on intestinal tissue maintenance and repair at conditions of stomach ulcers or at other clinical conditions like IBD.

IBD is a common denomination of ulcerative colitis (UC) and Crohn’s disease (CD), characterized by destructive intestinal inflammation, where wounds and injury in the gastrointestinal tract result in a disruption of the intestinal epithelial cell barrier function [12]. This may result in a continued exposure of factors, which potentially trigger the immune response. Therefore, it is important to rapidly reseal this barrier through stimulated ulcer healing. Furthermore, stomach ulcers can be observed in the gastro-intestinal tract as an unfortunate side effect in the treatment with widely described non-steroidal anti-inflammatory drugs (NSAIDs), which in some cases can cause mucosal erosions and asymptomatic ulcers in the gastro-intestinal tract.

The process of wound healing can be divided into three different and overlapping stages [13]. At first, within hours after injury, cells from the edge of the wound will migrate into the denuded area, a process called restitution. This is followed by cell proliferation, a stage that takes place within days after wounding, and which will proceed for several days or even months. When the wound gap is closed, the cells will go through the final stages of maturation, differentiation, and establishment of tight junctions between neighbouring epithelial cells to complete the healing and re-establish the cell wall barrier [14]. Therefore, natural bioactive components, including milk protein-derived peptides, with beneficial effects in relation to gastro-intestinal wound healing, have potential as oral supplements in relation to maintaining a healthy epithelial barrier and promote the wound healing process in several cases.

The aim of the present study is to investigate potential beneficial effects of bioactive peptides derived from casein by digestive enzymes on the initial part of epithelial cellular wound healing in the gastro-intestinal tract, using an in vitro cell model for wound healing, and to identify peptides present in active preparations using liquid chromatography–electrospray ionisation mass spectrometry (LC-ESI/MS/MS).

## 2. Materials and Methods

### 2.1. Casein Hydrolysates

Five g of spray-dried casein substrate (Sigma-Aldrich, Søborg, Denmark) was added to 500 mL of 0.05 M phosphate buffered saline (PBS) and 0.1 M NaCl at pH.7.4 (final concentration of casein was 10 g/L), and left for overnight stirring and dissolution at 4 °C. Then, 75 mL of each of dissolved casein substrate was heated in a water bath to 37 °C with stirring, pH was adjusted to 2.5 with 2 M HCl, and pepsin from porcine gastric mucosa (≥250 units/mg; Sigma-Aldrich, E.C. 3.4.23.1) was added to a final concentration of 0.08 g/L. After 1 h of pepsin digestion, the pH was adjusted to 7.4 with 2 M NaOH. Corolase PP (4000 U/g; AB enzymes, Darmstadt, Germany), a pancreatic protease preparation containing both endopeptidase (primarily trypsin and chymotrypsin) and exo-peptidase (amino- and carboxypeptidase) in addition to lipase activities, was added to a final concentration of 0.2 g/L, and digestion was continued at 37 °C for up to 2 h. Aliquot samples were obtained from these corolase digests at t = 0 min (taken before addition of corolase, representing only digestion with pepsin), 10, 30, 60, and 120 min, yielding a panel of individual casein hydrolysates. Control samples, without added enzyme, consisted of casein dissolved at 10 g/L in 0.05 M PBS and 0.1 M NaCl, pH 7.4, and heated in parallel in water bath at 37 °C with stirring, initially with pH adjusted to 2.5 with 2 M HCl for 1 h, and then subsequently adjusted to pH 7.4 with 2 M NaOH. Aliquots of control samples were taken at the same time points as for the digested samples. After incubation, one set of aliquots was used for Tris-Tricine gel analysis and mixed with 2 volumes of Tris-Tricine SDS-PAGE sample buffer (Biorad, Hercules, CA, USA) containing 0.2 M Tris-HCl, 40% *v*/*v* glycerol and 2% w/v SDS, boiled at 100 °C for 2 min and then stored at −20 °C until analyzed. Another parallel set of aliquots was used for in vitro assays of cell migration. A third set of aliquots was ultrafiltered using spin filters with 10 kDa cut-off (10 K filtrates) (Millipore, Ireland) at 14.000× *g* for 15 min at 4 °C, and further by 3 kDa cut-off value (3 K filtrates) (Millipore). The 10 K and 3 K filtrates were prepared from the casein hydrolysed with digestive enzyme for five different time points (*t* = 0, 10, 30, 60, and 120 min), giving a total of 10 samples for in vitro test of cell migration and cell proliferation (five time points for hydrolysis × two filtrations). These 10 K and 3 K filtrates, now all free of digestive enzymes, were stored at −80 °C until used in cell-based assays.

### 2.2. Tris-Tricine SDS-PAGE

The casein hydrolysates were analyzed by Tris-Tricine SDS-PAGE essentially as described earlier [15]. Tris-Tricine ready gels (10–20%) were obtained from Biorad and each well were loaded with 7 µL of hydrolysate (10 g/L) to be analyzed mixed with 14 μL Tris-Tricine sample buffer, giving a total of approximately 67 μg of protein loaded to each well. A protein marker (“broad”, Biorad, Copenhagen, Denmark) was used. The gels were stained with Coomassie Brilliant Blue G-250.

### 2.3. Free Amino Terminal Determination

The level of free primary amines in casein hydrolysates was determined according to the method of Larsen et al. [15]. Briefly, 50 µL of casein hydrolysate were mixed with 50 µL of 24% trichloroacetic acid and placed on ice for 30 min. The precipitate was removed by centrifugation (17,049× *g* at 4 °C for 20 min). A 37.5 μL aliquote of supernatant from each sample was added 1130 μL 0.1 M sodium tetraborate and 375 μL fluorescamine in water-free acetone (0.2 mg/mL). The mixture was shaken well. From each sample, 250 μL of sample was transferred into each of five wells in a microplate. After 18 min of incubation time with fluorescamine, the plates were measured on a luminescence spectrometer (LS50B, PerkinElmer, Waltham, MA, USA) with excitation at 390 nm and emission at 480 nm. Quantification was determined by calculating leucine equivalents using a leucine standard curve (0.5–3 mM leucine dissolved in 1 mM HCl) treated the same as the casein hydrolysates.

### 2.4. Cell Culture

Rat IEC-6 cells (DSMZ, Braunschweig, Germany) were cultured in 75 cm^2^ flasks in 10 mL DMEM (Invitrogen, Taastrup, Denmark) containing 10% fetal calf serum (FCS) (Lonza, Cologne, Germany), 0.1 M sodium pyruvate (Thermo Fisher, Hvidovre, Denmark), 0.2 M glutamax (Thermo Fisher), and with 1% penicillin/streptomycin (Sigma-Aldrich) added. Cells were kept in an incubator at 5% CO_2_ and 37 °C. The cell line has previously been used for measuring cell migration [10,16].

### 2.5. Cell Migration Assay

Cell migration was studied by a scratch assays in 12 well plates containing 1–2 × 10^5^ cells in each well essentially as described earlier [10,17]. Each well was scratched once to produce a cell-free area, and the IEC-6 cells were washed twice with PBS to remove free floating cells. Serum free growth medium (0% FCS) containing increasing concentrations of casein hydrolysates (0.1%, 1.0%, or 2.5%) were then added to two duplicate wells per concentration. Serum free medium (0% FCS) and medium with 10% FCS were added in duplicates to each plate as negative and positive control, respectively. A Leica DMIL microscope and a Leica digital camera were used to obtain photomicrographs, immediately after addition of the different hydrolysates and again 6 h later. Cell migration was measured by examining the width of the cell free area at each time point. Six measurements were achieved from each of two replicate wells. This assay was repeated twice, giving a total of 24 measurements of cell migration for each treatment medium.

### 2.6. Cell Proliferation Assay

Cell proliferation was studied with alamarBlue^®^ (BioSource, AH diagnostics, Aarhus, Denmark) as described earlier [18]. alamarBlue^®^ measures cell proliferation as viable cells metabolize resazurin into resorufin which is highly fluorescent. IEC-6 cells were cultured in 96-well plates, with 3000 cells/well in 200 µL medium containing 10% FCS for 24 h. After this, the cells were washed with PBS and added medium containing increasing concentrations of casein hydrolysates 10 K and 3 K filtrates (0.1%, 1%, 2%, and 5%). Serum free medium (0%) and medium with 10% FCS were added to each plate as negative and positive control, respectively. Cells were left in an incubator at 37 °C for 72 h, after which, 20 µL alamarBlue^®^ was added to each well. The fluorescence (excitation at 560 nm and emission at 590 nm) was measured after four, five, and six hours respectively. Six replicates of each concentration were measured.

### 2.7. Liquid Chromatography–Electrospray Ionization Tandem Mass Spectrometry Ion Trap Ion Trap Peptide Profiling

An Agilent 1200 Series capillary HPLC system (Agilent Technologies 2000, Waldbronn, Germany), consisting of a G1376A capillary pump and a G1377A autosampler, was used together with the Agilent Chemstation software as previously described with modifications [19]. Briefly, the 10 K and 3 K filtrates of the casein hydrolysates (1 µL) were injected onto a C18 RP-HPLC column and eluted using the following elution conditions of buffer A (0.1% formic acid) and buffer B (90% acetonitrile, 0.1% formic acid): 0–5 min 2% buffer B, 5–30 min 2–100% buffer B, 30–40 min 100% buffer B. Column temperature was 20 °C, and the flow rate was 10 µL/min. A Quadrupole ion trap tandem MS was used for mass analysis of separated peptides monitored at 214 nm and 280 nm. The spectrometer was operated in positive ion mode, and the following settings were used for the electrospray ionization interface parameters: capillary voltage 4 kV, nitrogen flow rate 5 L/min, capillary temperature 300 °C. Samples were analyzed with a full scan MS mode in the range 50–3000 m/z and total ion chromatograms were obtained. Then, the mass spectra corresponding to chromatographic peaks were analyzed using the Mascot search engine against a custom bovine milk protein database. Mass tolerance was set to 0.4 Da. Only peptides identified with a significant score *p* < 0.05 were included.

Identified peptides were further examined for homology with literature-identified bioactive peptide using the Milk Bioactive Peptide Database [3]. The search was conducted using the sequence search option and a similarity threshold was set to 100%.

### 2.8. Statistical Analysis

Analyses were carried out using the statistical program RStudio version 1.0.136. A general linear mixed model with Tukey’s HSD Post Hoc test was used to compare migration between cells treated with either casein hydrolysates or control samples. Level of significance was defined as *p* < 0.05.

## 3. Results

### 3.1. Casein Hydrolysate Analysis

**Figure 1:**
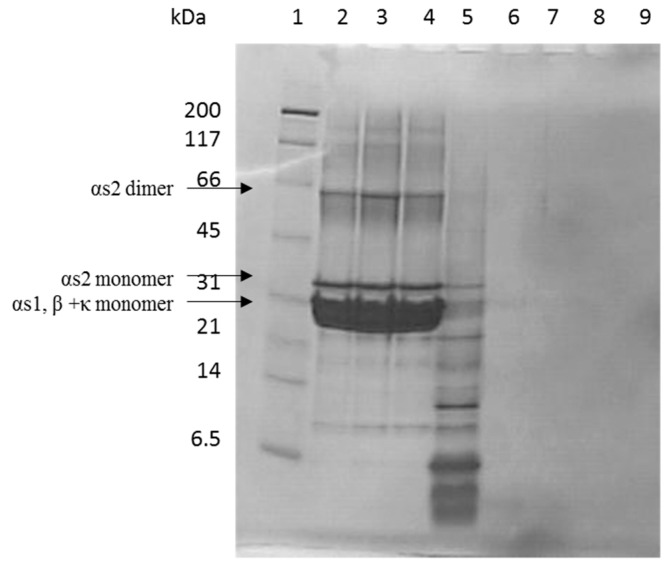
Tris-Tricine SDS-PAGE of casein hydrolysates. Casein was hydrolyzed at 37 °C for 1 h with pepsin followed by the addition of corolase. Lane 1: Molecular mass marker; lanes 2–4: Control casein incubated at 37 °C at pH 2.5 for 1 h followed by pH adjusted to 7.4 and subsequently incubated for 0, 60, or 120 min, respectively; lanes 5–9: casein incubated at 37 °C at pH 2.5 with pepsin for 1 h, followed by addition of corolase at pH 7.4 and incubated for *t* = 0, 10, 30, 60, 120 min, respectively. The gel was stained with Coomassie Brilliant Blue G-250. The positions of α_s1_ dimer, α_s2_ monomer, and α_s1_, β + κ monomers are shown on the gel.

The hydrolysis of casein into protein fragments and peptides by digestive enzymes was initially studied by Tris-Tricine SDS-PAGE (Figure 1). Hydrolysis of casein at *t* = 0 min with corolase, representing the 1 h treatment with pepsin only, resulted in a wide range of protein fragments and polypeptides, ranging in molecular masses from approximately 20 kDa to less than 6.5 kDa, in addition to a small amount of intact casein remaining (Figure 1, lane 4). Addition of corolase to the pepsin hydrolysed casein showed an extensive further hydrolysis, resulting in fragments generated mainly below the resolving range of the Tris-Tricine gel (well below 6.5 kDa) already after 10 min of hydrolysis with corolase (lane 6), where no bands were visible.

**Figure 2:**
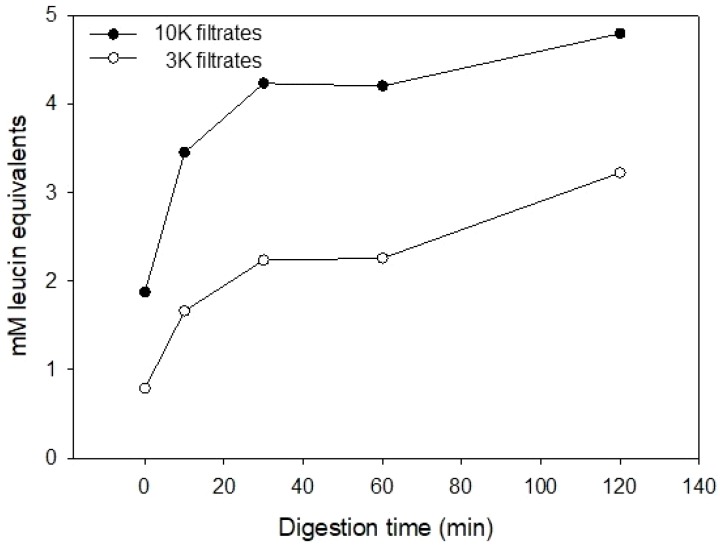
Free amino terminal determination of casein incubated at 37 °C at pH 2.5 with pepsin for 1 h, followed by addition of corolase at pH 7.4 and incubated for *t* = 0, 10, 30, 60, 120 min, respectively.

To further investigate the proteolysis of caseins by pepsin and pancreatic enzymes, the formation of free amino groups was determined at the different digestion timepoints (Figure 2). This was conducted after the hydrolysates had been filtrated by 10 kDa and 3 kDa filters. Both 10 K and 3 K casein hydrolysate revealed an increase in free amino groups, and thereby the formation of new peptides or free amino acids, or both. The concentration of free amino groups was higher in the 10 K filtrates than in the 3 K filtrates.

### 3.2. Effects of Casein Hydrolysates on Intestinal Cell Migration and Proliferation

After having confirmed the hydrolysis of the intact caseins by digestive enzymes, the casein hydrolysates were tested for wound healing activity by employing an in vitro assay for cell migration (scratch assay) using IEC-6 cells in culture. Initially, the casein hydrolysates were tested directly in the scratch assay without further fractionation at concentrations of 0.1%, 1.0%, and 2.5% in cell culture medium. It was evident that the presence of the digestive enzymes in the preparations of the casein hydrolysate samples affected the IEC-6 cells in a way so that they loosened from the culture plate surface (results not shown). Therefore, the casein hydrolysates further investigated were only the ultrafiltrated using spin filters with a 10 kDa cut-off, followed by spin filters with 3 kDa cut-off. By this ultrafiltration, casein peptides and fragments were separated from digestive enzymes and eventual undigested protein remnants, and peptides concentrated in the filtrate were used for further investigations. A total of 10 different casein hydrolysates were all investigated for their effect on cell migration at 0.1%, 1.0% and 2.5% concentrations in culture medium (Table 1).

The 10 K filtrates of casein hydrolysates incubated with pancreatic enzymes for 10 min stimulated migration with 36–45% for the three different concentrations (0.1%, 1.0%, and 2.5%) tested. The matching 3 K samples showed no stimulation of cell migration. The 10 K filtrates of casein hydrolysates digested for 30 min with pancreatic enzymes stimulated migration with 36–55% at a concentration of 0.1% and 1%. The matching 3 K filtrate samples only stimulated migration 22% at a concentration of 1%. Taken together, three of the ten hydrolysates appeared to stimulate cell migration to a significant degree.

Apart from cell migration, a potential effect of the prepared hydrolysates on cell proliferation was tested but showed no stimulatory effect (results not shown). This indicates that the bioactivity of the generated casein peptides in relation to wound healing was at the level of cell migration and not cell proliferation.

A high stimulatory effect of intestinal cell migration was observed for the 10 K filtrates of casein hydrolysates 10 min and 30 min and these were selected for peptide profiling, along with their corresponding 3 K filtrates. The 3 K showed lower or no stimulation of cell migration, but were selected for comparison of LC-ESI/MS/MS peptide profiles between highly active and less active casein hydrolysates in order to evaluate and compare peptide present in active versus less active casein hydrolysate filtrates, and from there draw conclusions on potential candidates for bioactive peptides stimulating intestinal cell migration in the employed in vitro scratch assay.

### 3.3. Identification of Peptides in Casein Hydrolysates by LC-ESI/MS/MS Ion Trap

The four casein hydrolysates selected (indicated in Table 1) for further investigation of their peptide profiles were subjected to LC-ESI/MS/MS ion trap analysis. In total, 130 different peptides were identified to be present in one or more of the four casein hydrolysates selected for peptide profiling (Supplementary Table S1). On average, 68.5 ± 2.1 peptides were identified in the two 10 K filtrate casein hydrolysates, while 49.0 ± 4.2 peptides were identified in the two 3 K filtrates. Next, the peptides were grouped according to their presence in either active (10 K filtrates 10 min and 30 min) or inactive/less active casein hydrolysates (3 K filtrates 10 min and 30 min). This information were used to indentify peptides that occured in the active casein hydrolysates, but not in any of the non-active/less-active casein hydrolysatess, given the rationale that peptides present in inactive casein hydrolysates would not be potential candidates for bioactivity.

Of the 130 identified peptides present in at least one of the casein hydrolysates investigated, 54 peptides were only identified in at least one of the active casein hydrolysates but not in the casein hydrolysates with no activity. Eleven of the 54 peptides were found to be present in both active casein hydrolysates. This information was used to generate a list of peptides (Table 2) hypothesized to be the most likely candidates for the observed bioactivity. Two derived from α_s1_-casein, one from β-casein (139–154), two from the region of β-casein 184–108, four from a specific region of β-casein region 158–178, and two from κ-casein.

### 3.4. Literature-Identified Bioactive Peptides

Searching the identified peptides against the database of milk bioactive peptides revealed that 15 unique peptides in the four casein hydrolysates were identical to previously reported bioactive peptides (Table 3). Six of these peptides are identical to known bioactive peptides with antimicrobial activity, while nine are known angiotensin-converting-enzyme inhibitors (ACE-inhbitors). Two of the peptides also showed antioxidant activity and one also had immuno-regulatory activity.

## 4. Discussion

In the present study, digestion of casein in the gastrointestinal tract was simulated by treatment with combinations of digestive enzymes at different pH values and for different time intervals. Pepsin was used at pH 2.5 for one hour, which matches the time of food transition through the stomach which has been reported to have a half time of 1.2 ± 0.3 h [20], and pH optimum of pepsin is around 2 [21]. The pancreatic enzymes mix was used at pH 7.4 from 0–2 h covering most of the transition time of the small intestine [22], which was reported to have a mean transition time of 4.0 ± 1.4 h, though food has already been observed to enter the colon after 2.8 ±1.5 h after ingestion [20]. This model of in vitro digestion are in accrodance with what have previously been used for in vitro digestion of dairy products [23].

The results from the employed in vitro cell migration assays showed that specific casein hydrolysates were active in stimulating cell migration. At short- (10 min) and medium-time (30 min) hydrolysis with pancreatic enzymes, a significant stimulatory effect was observed for the 10 K filtrates of the casein hydrolysates, while no or only small effect was measured in their corresponding 3 K filtrates. Long-time (60–120 min) hydrolysis produced casein hydrolysates with no stimulatory effects on intestinal cell migration. Together, this suggests that the active peptides may not survive exposure to pancreatic enzyme for more than 30 min. Furthermore, the active peptides could be retained by the 3 kDa spin-filter due to their size above this cut-off.

We previously tested the effect of commercial whey and casein preparations on wound healing mechanisms and found that the highest effect on cell migration was obtained with casein-based enzymatic hydrolysates [10]. However, a stimulation of cell migration by casein in vitro gastrointestinal digests as observed in this study has not previously been reported. Our result therefore complements previous findings and indicate that a stimulatory effect on cell migration can also be obtained during digestion of intact caseins by gastrointestinal enzymes.

The integrity of the intestinal mucosal surface is rapidly re-established after injury by three overlapping processes. First, cells from the edge of the wound migrate into the exposed cell-free area followed by proliferation to close the wound gap. The final process is cell maturation, differentiation, and re-establishing of the epithelial surface. The results presented in this study show that casein digests could stimulate the initial important migration process of wound healing. Since the bioactivity observed was released during digestion of casein with pancreatic enzymes from 10–30 min, the small intestine is considered the relevant site of action. The result here could therefore be relevant for wound healing in clinical conditions like IBD, especially for CD.

We previously found that commercial casein and whey enzymatic preparations increased cell proliferation in IEC-6 cells [10], although quite high milk protein concentrations (>500 µg/mL) were required for stimulation of proliferation of 20% or more after an incubation period of 72 h. However, in the present study the casein digests had no effect on cell proliferation. It could be speculated that the concentration required for stimulation of proliferation was above the concentration of casein hydrolysates used here. In addition, it would be interesting to see whether the result would have been different if proliferation was measured after injury to the cell layer similar to the procedure for the cell migration assay (scratch assay). Future studies should therefore further explore this effect.

The peptide profiles of selected casein hydrolysates were investigated by LC-ESI/MS/MS Ion Trap analysis of both 10 K and 3 K filtrates of the same hydrolysates to obtain knowledge of the correlation and variation between the peptide profile of the 10 K and 3 K filtrates. The number of identified peptides between 10 K and 3 K samples and flourescamine assay confirmed that peptides were excluded by the 3 kDa cut-off filter. By analyses of the results and by comparing peptides identified in different casein hydrolysates, we obtained knowledge about which peptides are plausibly present in the 10 K filtrates with effect and without in their matching 3 K filtrates without effect. Eleven peptides were identified meeting these criteria and are therefore good candidates for the bioactivity observed in migration of intestinal epithelial cells. Two derived from α_s1_-casein, seven from β-casein, and two from κ-casein. When searching for likely candidates that cause the desired bioactivity, it is important to consider that it could be generated from a synergy between different peptides. A previous study supports the idea that a synergy between components can cause the highest effect [24].

Besides the observed bioactivity, the total pool of 130 unique peptides identifed in the four casein hydrolysates contained 15 peptides, with identity to already known bioative peptides. However, the peptides identified in the present study representing the most likely candidates for causing the observed stimulation in cell migration have not previously been identified as bioactive according to the MBPDB [3].

Several of the peptides proposed as responsible for the bioactivity observed on intestinal cell migration had overlapping parts with other known bioactive peptides [3]. The peptide α_s1_-casein (189–208) contains an antimicrobial region α_s1_-casein (195–208) [25]. The petide β-casein (139–154) contained the region from 146–154 in which several ACE-inhibitory peptides has previously been identified [26]. Two peptides deriving from β-casein, (representing residues 84–108 or 96–107) showed a high degree of sequence similarity (≥80%) to the sequence of known ACE-inhibitory [27] and antimicrobial [28] peptide. The peptide from κ-casein (70–81) further showed a high degree of similarity to the sequence of a known dipeptidyl peptidase-4 inhibitory peptide [29].

The LC-ESI/MS/MS analysis showed a widespread peptide profile of the casein hydrolysates, and data analysis suggested specific peptides to be responsible for the bioactivity related to migration of epithelial cells. However, further studies with these potential beneficial peptides in animal models and human studies are needed in order to develop commercial products for wound healing in the gastrointestinal tract.

## 5. Conclusions

In conclusion, this study showed that peptides derived from bovine casein hydrolysed with gastronintestinal digestive enzymes have the ability to stimulate cell migration of intestinal epithelial cells in vitro, which is an important step in gastro-intestinal wound healing. Eleven peptides were potential candidates for the stimulating effect on cell migration as these were present in digestive samples with high activity on cell migration and not in those without activity. These peptides derived from αs1-casein, κ-casein, and specific regions of β-casein. Amongst the identified peptides from the gastro-intestinal digestion of casein we found that 15 of these had previously been reported to exert antioxidant, antimicrobial, ACE-inhibitory, and immunomodulatory activity. The observed bioactivity in the in vitro assay employed could therefore potentially exert a wound healing effect on epithelial cell lining in the innermost layer of the gastro-intestinal tract upon ingestion of casein, but this would need further in vivo studies to be verified.

## Figures and Tables

**Table 1:** The effect of 10 K and 3 K filtrates of casein hydrolysates on cell migration (*n* = 24).

		Casein Hydrolysate ^a^			
	Time Point (min)	0.0%	0.1%	1.0%	2.5%
10 K	0	1.00 ± 0.13	1.20 ± 0.13	1.12 ± 0.13	1.01 ± 0.14
	10 ^b^	1.00 ± 0.16	1.42 ± 0.15 **	1.36 ± 0.15 *	1.45 ± 0.17 **
	30 ^b^	1.00 ± 0.20	1.36 ± 0.18 *	1.55 ± 0.17 **	1.15 ± 0.17
	60	1.00 ± 0.14	0.91 ± 0.14	1.13 ± 0.13	0.96 ± 0.14
	120	1.00 ± 0.12	1.18 ± 0.12	1.10 ± 0.12	0.77 ± 0.18
3 K	0	1.00 ± 0.13	1.01 ± 0.14	0.97 ± 0.15	0.98 ± 0.13
	10 ^b^	1.00 ± 0.12	1.08 ± 0.10	1.09 ± 0.12	1.09 ± 0.12
	30 ^b^	1.00 ± 0.10	1.14 ± 0.10	1.22 ± 0.10 **	1.05 ± 0.10
	60	1.00 ± 0.14	1.19 ± 0.15	1.23 ± 0.05	1.13 ± 0.19
	120	1.00 ± 0.11	1.10 ± 0.11	1.00 ± 0.1	0.94 ± 0.10

^a^ Values are presented as relative to cell migration obtained in basal medium without any addition of casein hydrolysates (BM = 1.00) and given as means ± SEM. * *p* < 0.05, ** *p* < 0.01. ^b^ The peptide profile of these samples was further investigated by LC-ESI/MS/MS.

**Table 2:** Peptides identified as potential candidates for intestinal cell migration activity due to their differential presence in active and non-active casein hydrolysates as determined by LC-ESI/MS/MS.

Protein	Mass [M + H]^+^	Peptide Sequence
α_s1_-casein (148–158)	1200.6	EPMIGVNQELA
α_s1_-casein (189–208)	2105.0	TDAPSFSDIPNPIGSENSEK
β-casein (139–154)	1775.0	SLTLTDVENLHLPLPL
β-casein (158–171)	1698.8	WMHQPHQPLPPTVM
β-casein (158–177)	2354.2	WMHQPHQPLPPTVMFPPQSV
β-casein (158–178)	2467.2	WMHQPHQPLPPTVMFPPQSVL
β-casein (160–171)	1381.7	HQPHQPLPPTVM
β-casein (84–108)	2741.5	SLPQNIPPLTQTPVVVPPFLQPEVM
β-casein (96–107)	1320.8	PVVVPPFLQPEV
κ-casein (134–145)	1373.7	NQDKTEIPTINT
κ-casein (70–81)	1452.8	ALINNQFLPYPY

**Table 3:** Bioactive peptides identfied in casein hydrolysates by searching the milk bioactive peptide database (MBPDB).

Protein	Sequence	Function
α_s1_-casein (39–48)	FVAPFPEVFG	ACE-inhibitory ^a^
α_s1_-casein (119–124)	YKVPQL	ACE-inhibitory
α_s1_-casein (195–208)	SDIPNPIGSENSEK	Antimicrobial
α_s2_-casein (96–104)	ALNEINQFY	ACE-inhibitory
α_s2_-casein (204–212)	AMKPWIQPK	ACE-inhibitory
β-casein (74–83)	VYPFPGPIPN	ACE-inhibitory, antioxidant
β-casein (113–120)	VKEAMAPK	Antimicrobial, antioxidant
β-casein (115–120)	EAMAPK	Antimicrobial
β-casein (121–128)	HKEMPFPK	Antimicrobial
β-casein (185–191)	VLPVPQK	Antimicrobial, ACE-inhibitory, antioxidant
β-casein (208–224)	YQEPVLGPVRGPFPIIV	ACE-inhibitory, immunoregulatory
β-casein (209–224)	QEPVLGPVRGPFPIIV	ACE-inhibitory
β-casein (208–222)	YQEPVLGPVRGPFPI	Antimicrobial
κ-casein (72–81)	INNQFLPYPY	ACE-inhibitory
κ-casein (46–51)	YIPIQY	ACE-inhibitory

^a^ angiotensin-converting-enzyme inhibitory (ACE-inhibitory).

## Supplementary Materials

The following are available online at https://www.mdpi.com/2304-8158/8/3/91/s1, Table S1: Peptides identified in casein hydrolysates by LC-ESI/MS/MS.
